# Chemical compound identification and antibacterial activity evaluation of cinnamon extracts obtained by subcritical n‐butane and ethanol extraction

**DOI:** 10.1002/fsn3.1065

**Published:** 2019-05-16

**Authors:** Ying Liang, Yi Li, Aidong Sun, Xianjin Liu

**Affiliations:** ^1^ Key Laboratory of Food Quality and Safety of Jiangsu Province, Institute of Food Safety and Nutrition Jiangsu Academy of Agricultural Science Nanjing China; ^2^ School of Food and Biological Engineering Jiangsu University Zhenjiang China

**Keywords:** antibacterial activity, cinnamon, GC‐MS, HPLC‐MS, subcritical extraction

## Abstract

Four important *Cinnamomum* species in China including *C. cassia*, *C. loureiroi*, *C. wilsonii*, and *C. burmannii* were chosen to be extracted by subcritical n‐butane and ethanol. The chemical compounds of extracts were identified by GC‐MS and HPLC‐MS, and the antibacterial activities were evaluated by agar‐well diffusion assay and twofold microdilution broth method. There were 47 compounds identified in n‐butane extracts and 11 compounds in ethanol extracts totally. The major compounds in n‐butane extracts varied significantly among different *Cinnamomum* species, and (*E*)‐cinnamaldehyde and coumarin were major compounds for *C. cassia* with area percentage of 74.32%; (*E*)‐cinnamaldehyde and α‐copaene for *C. loureiroi* with area percentage of 67.83%; linalool, (*E*)‐cinnamaldehyde, and citral for *C. wilsonii* with area percentage of 58.74%; and eugenol, (*E*)‐cinnamaldehyde, and coumarin for *C. burmannii* with area percentage of 76.43%. The maximum compounds in ethanol extracts were (*E*)‐cinnamaldehyde and (*Z*)‐cinnamaldehyde, and others varied among the *Cinnamomum* species. All cinnamon extracts showed antibacterial activities that n‐butane extracts were much more sensitive than ethanol extracts. The inhibition zone for N‐butane extracts against *Listeria monocytogenes*, *Staphylococcus aureus*, *Escherichia coli*, and *Salmonella anatum* was from 18.98 to 37.45 mm while for ethanol extracts from 7.11 to 10.11 mm. The minimum bactericidal concentrations for n‐butane extracts were ranged from 0.31 to 2.50 mg/ml and for ethanol extracts ranged from 20.00 to 160.00 mg/ml. N‐butane extracts of *C. cassia* and *C. loureiroi* processed much higher antibacterial activities than *C. wilsonii* and *C. burmannii.* N‐butane extracts of *C. cassia* and *C. loureiroi* have the potential to be used as food biopreservative.

## 1 INTRODUCTION

The genus *Cinnamomum* comprises over 250 species cultivated commercially in tropical and subtropical regions of China, India, South America, and Africa (Jayaprakasha & Rao, 2011; Wang, Wang, & Yang, 2009). It has been proved that the cinnamon has anti‐inflammatory, antimicrobial, antioxidant, cardiovascular, and immunomodulatory effects according to in vitro and in vivo evidence (Gruenwald, Freder, & Armbruester, 2010). So, as spice and traditional herbal medicine, cinnamon has been used widely for thousands of years in many countries, especially in China.

*Cinnamomum cassia*, which is named Chinese cinnamon and originated in southern China, has been cultivated widely in Guangxi and Guangdong provinces. *C. loureiroi*, known as Vietnamese cinnamon, is indigenous in Southeast Asia. *C. burmannii*, also called Indonesian cinnamon which is native to Southeast Asia, is a common and cheap type to make powder. *C. cassia*, *C. loureiroi*, and *C. burmannii* are important species in regional markets especially in China (Jayaprakasha & Rao, 2011). *C. wilsonii*, which is endemic to China, is widely distributed in Sichuan, Hubei, Guangxi, and Guangdong provinces of China (Wang et al., 2009).

Foodborne illness outbreaks are common and often cause considerable morbidity throughout the world in recent years (Havelaar et al., 2015). Natural substances of plant origin which have good antibacterial activities could be excellent resources to control microbial growth and reduce the incidence of food poisoning and spoilage. *Cinnamomum* species have long been used in food not only for their flavor but also for their preservative properties (Geng et al., 2011). Cinnamon bark essential oil, as one of the most important products of cinnamon, has been studied widely on compound compositions and antibacterial activities against foodborne pathogens. Nevertheless, information regarding the different *Cinnamomum* species is still limited. The essential oil of *C. cassia* (Geng et al., 2011; Huang, Xu, Liu, Zhang, & Hu, 2014; Li, Kong, & Wu, 2013) and *C. burmannii* (Awang, Susanti, & Taher, 2013; Chairunnisa, Tamhid, & Nugraha, 2017) has been researched on compound compositions and antibacterial activities, and *C. loureiroi* (Li, Wang, Jiang, & Jiang, 2010) and *C. wilsonii* (Tao, Sun, & Ding, 2002) only on compound compositions according to the literature. The evaluation of antibacterial activities of cinnamon extracts would be useful for their applying in the prevention of food spoilage and deterioration and also in extension shelf life of foods.

Essential oil was extracted mostly by water distillation, if different polar solvents are used for bioactive compounds extraction, and subsequent solvent partition allows finer division into different polarity fractions. Subcritical extraction is an excellent extraction that has numerous advantages such as lower operating temperature and pressure, shorter extraction time, and environmental compatibility (Herrero, Cifuentes, & Ibanez, 2006). It can be used with different polarity subcritical fluids and considered a technological revolution in the extraction industry. Subcritical extraction used in cinnamon was researched only by Pramote et al. (2012), who extracted the *C. zeylanicum* using subcritical water and found the components might degrade during subcritical water treatment because of high temperature. Better results are more likely to get using different polar subcritical solvent with lower temperature in cinnamon extraction. N‐butane is used mainly as low‐polarity solvent because of excellent dissolving power for lipophilic components, and it needs lower subcritical pressure and temperature (Herrero et al., 2006). Ethanol is a high polar solvent and widely used as subcritical solvent for more polar compound extraction and relatively safe for human (Shi et al., 2005). Subcritical temperature of ethanol is much lower than water. In this project, four important *Cinnamomum* species in China including *C. cassia*, *C. loureiroi*, *C. wilsonii*, and *C. burmannii* were chosen to be extracted by subcritical n‐butane and ethanol, the chemical compositions of cinnamon extracts were identified, and antibacterial activities to four foodborne pathogens were evaluated to get more comprehensive information.

## 2 MATERIALS AND METHODS

### 2.1 Plant materials

*Cinnamomum cassia*, *C. loureiroi*, and *C. burmannii* barks were collected from the trees with the age from 10 to 15 in Guangxi province of China. *C. wilsonii* barks were obtained from the trees with the age of 11 in Sichuan province of China. The collected samples were ground to fine powder by a cutting mill and pass through a 50 mesh screen. The ground samples were stored at −20°C until used.

### 2.2 Subcritical extraction of plant materials

Four *Cinnamomum* species were extracted with n‐butane and ethanol separately using subcritical extraction methods. The extractions were conducted in a 5‐L extraction vessel. The parameters of subcritical n‐butane extraction were as follows: extraction time 30–50 min, temperature 30–50°C, and solvent‐to‐solid ratio 1–3. The parameters of subcritical ethanol extraction were as follows: extraction time 50–70 min, temperature 120–160°C, and solvent‐to‐solid ratio 12–16. The extracts were filtered and removed solvent by using rotary vacuum evaporator at 40°C. The resulting extracts were kept at 4°C for further analysis. The percentage of extracts yield was calculated as the weight of extracts divided by the weight of bark powder.

### 2.3 Component analysis of cinnamon extracts

#### 2.3.1 GC‐MS conditions

The n‐butane extracts were diluted in methanol and analyzed using Agilent GC System 6890 Series coupled to Agilent 5973 Network Mass Selective Detector. Samples were injected with an Agilent 7683 Series Autosampler. The injector temperature was 260°C, and injection was in splitless mode. The HP5‐MS column (60 m × 0.25 mm, 0.25 µm film thickness) was used with helium as a carrier gas (99.999% purity) at a flow rate of 1 ml/min. The GC oven temperature was raised from 75 to 190°C at the rate of 10°C/min, then raised to 280°C at the rate of 20°C/min, and held for 5 min. The temperature of transfer line and ion source was 260 and 200°C, respectively. Screening of the chromatograms was performed in scan mode from *m*/*z* 40 to 450 at a rate of 6.61 uma/s. Component identification was accomplished matching the mass spectra with standards from the Wiley^®^ 275.L library. Quantitative analyses of components of interest expressed as area percentage were carried out by a peak area normalization measurement, three injections for each sample.

#### 2.3.2 HPLC‐MS conditions

The ethanol extracts were analyzed using Agilent 1100 Series LC/MSD Trap with an electrospray ion source (ESI). All data were acquired employing Agilent Quantitative Analysis data processing software. Chromatographic separation was achieved by gradient elution using Polaris C_18_‐A column (250 × 4.6 mm) (Varian). The mobile phase consisted of solvent A (0.1% formic acid in water) and solvent B (0.1% formic acid in methanol). The gradient program was as follows: 0–20 min, 50% B; 20–35 min, 50%–80% B; and 35–45 min, 80% B. The flow rate was 1.0 ml/min. The UV detector was set at 280 nm. The LC elute was introduced directly into the ESI interface without flow splitting. The scan range of ESI‐MS was *m*/*z* 120–1,200. The ESI voltage was 4.5 kV in positive ion mode. The velocity of 9 L/min and temperature of 325°C of drying gas were applied for ionization using nitrogen. The nebulizer pressure was 40 psi. Relative percentage amount of major components was calculated according to individual peak area and total peak area of LC chromatogram as mean values of three injections from each sample.

### 2.4 Antibacterial activities of cinnamon extracts

#### 2.4.1 Microorganisms and culture

*Listeria monocytogenes*, *Staphylococcus aureus*, *Escherichia coli* O157:H7, and *Salmonella anatum* strains were utilized in this study as representatives of gram‐positive and gram‐negative pathogenic bacteria. The strains were cultured at 37°C on tryptic soy agar (TSA) medium.

#### 2.4.2 Agar‐well diffusion method

Agar‐well diffusion method was applied for the determination of antibacterial activity. All the bacterial strains were suspended in sterile physiological saline and diluted to the density of 1 × 10^6 ^CFU/ml. The suspension of 100 μl was spread onto the surface of TSA medium. 4.6‐mm wells in diameter were cut from the agar, and 50 μl sample solutions were delivered into them. 5 mg/ml kanamycin was used as positive reference standard to determine the sensitivity of each microbial species. Negative controls were prepared using PBS solution. The inoculated plates were incubated at 37°C for 24 hr. Antibacterial activity was evaluated by measuring the diameter of inhibition zone (DIZ) surrounding the wells. DIZ was expressed in millimeters. Tests were performed in triplicate.

#### 2.4.3 Twofold microdilution broth method

It is difficult to detect the minimum inhibitory concentration (MIC) precisely by the unaided eyes because the cinnamon extracts are brown. Dilutions were used to dispense 0.1 ml into each of the sterile 96 wells of a standard tray. Each well contained 5 × 10^5^ CFU/ml of test bacteria, serially diluted test samples, and tryptic soy broth (TSB) medium. The positive control well contained inoculated medium without test samples. Microdilution trays were incubated at 37°C for 20 hr in an ambient air incubator. For determination of minimum bactericidal concentration (MBC), 100 μl samples were taken from wells of microdilution trays and spread on freshly prepared TSA plates, and then incubated at 37°C for 24 hr to determine the MBC. The MBC was regarded as the lowest concentration of the samples that allowed <0.1% of the original inoculum treated with the extract or compound samples to survive and grow on the surface of the medium used. Triplicate samples were performed for each test concentration.

### 2.5 Statistical analysis

Quantitative analyses of components expressed as area percentage in GC‐MS and HPLC‐MS were calculated as mean values of three injections from each sample ± standard deviation. Chromatographic features were considered statistically relevant when *p* < 0.05. The results of all DIZ values were calculated as mean values ± standard deviations. Statistical analysis was performed using SPSS 10.0 computer software package.

## 3 RESULTS AND DISCUSSION

### 3.1 Yield of cinnamon extracts

The parameters of subcritical extraction were optimized to get higher yield. The optimal conditions of subcritical n‐butane extraction were as follows: extraction time 45 min, extraction temperature 40°C, and liquid‐to‐solid ratio 2. The optimized parameters of subcritical ethanol extraction were as follows: extraction time 60 min, extraction temperature 150°C, and solvent‐to‐solid ratio 15. Extraction solvent and *Cinnamomum* species are major factors that influence the yield. The yields of n‐butane extracts for *C. cassia* and *C. loureiroi* were 3.45% and 2.95%, and for *C. wilsonii* and *C. burmanni* were 1.89 and 1.56%, respectively*.* The yields of ethanol extracts for *C. cassia* and *C. loureiroi* were 12.49 and 13.57%, and for *C. wilsonii* and *C. burmanni* were 9.93 and 8.45%, respectively*.* The yields of ethanol extract were much higher than n‐butane extract, which could be due to higher polarity of ethanol and also higher extraction temperature. More extracts of *C. cassia* and *C. loureiroi* were got than *C. wilsonii* and *C. burmanni*. It is difficult to compare the yield with the literature. According to Geng et al. (2011), the yield of *C. cassia* essential oil extracted by hydrodistillation varied within 0.41%–3.11%; not only the extraction method and *Cinnamomum* species, but also the age and segment (top, center, and lower) of the tree influence the yield.

### 3.2 Compositions of n‐butane extracts

The subcritical n‐butane extracts obtained from *Cinnamomum* species were analyzed by GC‐MS; 47 compounds were identified totally in four extracts and are shown in Table 1. The compounds including alcohols, aldehydes, esters, carboxylic acids, alkanes, and ketones varied significantly among the different *Cinnamomum* species.

**Table 1:** The chemical compounds identified from the subcritical n‐butane extracts

Compound	Area percentage (%)			
	*C. cassia*	*C. loureiroi*	*C. wilsonii*	*C. burmannii*
Styrene	0.13 ± 0.07	−	0.02 ± 0.02	0.30 ± 0.11*
Benzaldehyde	0.21 ± 0.11	0.33 ± 0.20	0.34 ± 0.17	0.12 ± 0.11
Camphene	0.30 ± 0.21	0.11 ± 0.10	0.53 ± 0.13	0.13 ± 0.03
α‐Pinene	0.05 ± 0.04	−	0.03 ± 0.02	0.08 ± 0.06
β‐Pinene	0.07 ± 0.01	−	0.12 ± 0.09	0.14 ± 0.10
Myrcene	−	−	0.28 ± 0.19	−
p‐Cymene	−	−	0.49 ± 0.08*	−
Limonene	−	−	1.34 ± 1.10	−
1,8‐Cineole	−	−	5.54 ± 0.09*	−
Linalool	0.52 ± 0.10	−	23.66 ± 3.25*	−
Linalyl acetate	−	−	0.07 ± 0.05	−
Borneol	0.22 ± 0.08	0.25 ± 0.19	2.11 ± 0.08*	3.28 ± 0.09*
Terpinen‐4‐ol	0.10 ± 0.09	−	−	−
α‐Terpineol	0.21 ± 0.13	−	0.45 ± 0.18	0.85 ± 0.48
(*Z*)‐Cinnamaldehyde	1.23 ± 0.35	0.20 ± 0.18*	3.25 ± 1.55	1.27 ± 0.79
(*E*)‐Cinnamaldehyde	62.96 ± 4.21	51.69 ± 5.66*	19.63 ± 2.02*	34.44 ± 3.76*
α‐Copaene	3.78 ± 1.08	16.14 ± 3.46*	−	−
Cinnamaldehyde dimethyl acetal	−	5.66 ± 1.65*	−	−
α‐Cubebene	−	0.13 ± 0.09	−	−
(E)‐Cinnamyl acetate	0.40 ± 0.16	−	8.65 ± 0.07*	−
Camphor	0.95 ± 0.11	−	−	−
Carvone	−	−	−	0.12 ± 0.10
Methoxyacetophenone	−	−	−	0.22 ± 0.18
Citral	−	−	15.45 ± 3.78*	−
α‐Guaiene		3.19 ± 1.97	−	
α‐Humulene	−	0.48 ± 0.30		−
α‐Muurolene	0.34 ± 0.20	4.78 ± 1.32*		−
Geranyl acetate	0.15 ± 0.11	−	2.87 ± 1.95	−
Nerol	−	−	0.03 ± 0.03	−
Geraniol	0.42 ± 0.19	−	0.32 ± 0.11	−
Neral	−	−	0.95 ± 0.09*	
Eugenol	0.09 ± 0.08		0.22 ± 0.20	25.67 ± 3.85*
2‐Propenal	−	0.12 ± 0.12	−	−
3‐Methoxy‐1,2‐propanediol	3.26 ± 0.25		−	−
1,2,4‐Metheno‐1H‐indene	−	0.37 ± 0.31	−	−
β‐Cadinene	−	5.19 ± 0.28*	−	−
Naphthalene	0.13 ± 0.11	0.52 ± 0.31	−	−
α‐Calacorene	0.08 ± 0.06	0.26 ± 0.14	−	−
Cadina‐1,4‐diene	−	1.52 ± 0.97	−	−
Germacrene	−	0.29 ± 0.20	−	−
α‐Amorphene	−	1.25 ± 1.00	−	
Coumarin	11.36 ± 1.14	0.25 ± 0.09*	−	16.82 ± 4.66
Ethyl cinnamate	0.06 ± 0.04	−	−	0.22 ± 0.17
Methyl cinnamate	0.34 ± 0.22	−	1.05 ± 0.02*	3.16 ± 1.11*
β‐Caryophyllene	−	0.35 ± 0.15*	0.32 ± 0.07*	−
β‐Patchoulene	−	1.80 ± 1.25	−	−
α‐Elemene	−	0.05 ± 0.05	−	−
Total	90.55 ± 2.61	91.74 ± 4.21	87.72 ± 7.47	86.82 ± 3.54*
Number of compounds	25	22	24	15

* *p* value <0.05 when compared with *C. cassia* by *t* test.

As shown in Table 1, 25 compounds were identified and represented 90.55% of total peak area in *C. cassia* extract, (*E*)‐cinnamaldehyde was confirmed to be the major component with the highest area percentage of 62.96%, and other main components included coumarin, α‐copaene, 3‐methoxy‐1, 2‐propanediol, and α‐guaiene with area percentages of 11.36%, 3.78%, 3.26%, and 3.19%, respectively. Twenty‐two compounds accounted for 91.74% of the total peak area were identified in *C. loureiroi* extract, and (*E*)‐cinnamaldehyde (51.69%) was the leading compound, followed by α‐copaene (16.14%), cinnamaldehyde dimethyl acetal (5.66%), β‐cadinene (3.19%), and α‐muurolene (4.78%). *C. wilsonii* extract contains 24 compounds accounting for 87.72% of the total peak area. Linalool (23.66%), (*E*)‐cinnamaldehyde (19.63%), citral (15.45%), (*E*)‐cinnamyl acetate (8.65%), and 1, 8‐cineole (5.54%) as the major compounds were identified by GC‐MS. There were only 15 compounds accounting for 86.82% of peak area detected from *C. burmannii* extract. The major compounds were in decreasing order of (*E*)‐cinnamaldehyde (34.44%) > eugenol (25.67%) > coumarin (16.82%) > borneol (3.28%) > methyl cinnamate (3.16%).

The major components of extracts from *Cinnamomum* species were different from each other. (*E*)‐cinnamaldehyde was one of the most important components occurring in four *Cinnamomum* species, especially in *C. cassia*, *C. loureiroi*, and *C. burmannii*. Linalool and citral were main compounds only in *C. wilsonii* and eugenol in *C. burmannii*. Coumarin was also important in *C. cassia* and *C. burmannii*. Some quantitative and qualitative differences were found in our study and other reports of cinnamon oils. Huang et al. (2014) and Li et al. (2013) reported (*E*)‐cinnamaldehyde was a major component and accounted for 66.28%–77.21% in *C. cassia* barks oil and 81.97% in *C. loureiroi* bark oil. Chairunnisa et al. (2017) and Shan, Cai, Brooks, and Corke (2007) found (*E*)‐cinnamaldehyde was predominant component in *C. burmannii* bark oil but with no eugenol was detected. According to the study of Tao et al. (2002), (*E*)‐cinnamyl acetate (21.14%), (*E*)‐cinnamaldehyde (16.46%), and linalool (7.65%) were major components in *C. wilsonii* bark oil. The sequence of major components in *C. wilsonii* bark oil was different from our research. These results point out the method of extraction influencing the composition of extracts. Although in the same species, a variation of the compounds can be found in cinnamon plant which grows in different places (Guenther, 2006). Still, the major components in the same species are almost the same with different quantity.

### 3.3 Compositions of the ethanol extracts

HPLC‐MS was used to identify the ethanol extracts which contained high levels of nonvolatile compounds. Eleven compounds were identified totally according to the retention time and UV spectra of available authentic standards or by comparison of MS data and literature data (Maatta‐Riihinen, Kahkonen, Torronen, & Heinonen, 2005). The components in four ethanol extracts of *Cinnamomum* species are listed in Table 2.

**Table 2:** The chemical compounds identified from the subcritical ethanol extracts

Compound	MS (*m*/*z*) [M+H]^+^	Area percentage (%)			
		*C. cassia*	*C. loureiroi*	*C. wilsonii*	*C. burmannii*
Procyanidin B1	579	0.29 ± 0.20	0.03 ± 0.03	5.77 ± 1.56*	0.04 ± 0.03
Procyanidin B2	579	0.03 ± 0.02	0.04 ± 0.02	0.13 ± 0.12	4.06 ± 1.41*
Procyanidin trimer	865	1.96 ± 1.05	0.23 ± 0.21*	16.09 ± 3.46*	11.98 ± 5.37*
Catechin	290	0.01 ± 0.01	0.83 ± 0.65	0.92 ± 0.05*	1.65 ± 0.05*
Procyanidin dimer	577	−	0.12 ± 0.11	0.24 ± 0.12*	0.24 ± 0.03*
Epicatechin	290	−	0.51 ± 0.21*	0.09 ± 0.01*	0.01 ± 0.01
Coumarin	147	5.26 ± 2.35	1.97 ± 0.09	7.31 ± 4.12	6.29 ± 1.76
(*E*)‐Cinnamic acid	149	−	−	−	1.64 ± 0.06
(*E*)‐Cinnamaldehyde	133	67.92 ± 6.45	72.53 ± 4.69	42.31 ± 5.26*	48.48 ± 2.25*
(*Z*)‐Cinnamaldehyde	133	11.13 ± 3.24	10.09 ± 3.15	10.17 ± 1.23	14.28 ± 1.57
Cinnamyl alcohol	135	0.41 ± 0.35	3.64 ± 0.05*	6.06 ± 1.36*	3.98 ± 0.08*
Total		87.01 ± 4.32	89.99 ± 5.87	89.09 ± 3.47	92.65 ± 2.79*
Number of compounds		8	10	10	11

* *p* value <0.05 when compared with *C. cassia* by *t* test.

Proanthocyanidins, naturally occurring compounds, were found highly in cinnamon bark, especially in *C. wilsonii* and *C. burmannii* with peak area percentages of 21.99% and 12.08%, respectively. Coumarin and cinnamyl alcohol were also major compounds with peak area percentages of 1.97%–7.31% and 0.41%–6.06%. (*E*)‐Cinnamaldehyde and (*Z*)‐cinnamaldehyde processing peak area percentages of 52.48%–82.62% were most important compounds in ethanol extracts. The compositions of ethanol extracts were different among each other, but the major compounds were not varied significantly as n‐butane extracts.

Previous reports on *Cinnamomum* spices mainly focused on the essential oils, but fewer reports studied the nonvolatile components. He et al. (2005) used methanol as extraction solvent and RP‐HPLC to determine the components of cinnamon bark extracts, and only four characteristic components including cinnamaldehyde, cinnamic acid, cinnamyl alcohol, and coumarin were found. Shan et al. (2007) extracted the *C. burmannii* with 80% methanol, and 10 compounds, mainly (*E*)‐cinnamaldehyde and proanthocyanidins, were identified by LC‐MS. Gu et al. (2004) also reported that cinnamon contained very high levels of proanthocyanidins. The major components in polar extract of *Cinnamomum* spices were basically in accordance with our study.

### 3.4 Antibacterial activity of the cinnamon extracts

The aim of the present study was to assess the antibacterial activities of cinnamon extracts and to compare their effectiveness against four foodborne pathogens (two gram‐positive and two gram‐negative) by agar‐well diffusion assay and twofold microdilution broth method. The inhibition zone of the cinnamon extracts is summarized in Table 3. The inhibition zone above 5 mm in diameter was taken as positive result. The results revealed that all the n‐butane extracts and ethanol extracts exhibited antibacterial activities to gram‐positive and gram‐negative pathogens with varying values. N‐butane extracts of cinnamon showed significant inhibitory effect against *L. monocytogenes*, *S. aureus*, *E. coli*, and *S. anatum* with inhibition zone from 18.98 to 37.45 mm. Ethanol extracts of cinnamon were potentially active against four foodborne pathogens with inhibition zones ranging from 7.11 to 10.11 mm. Both n‐butane extracts and ethanol extracts showed no significant differential between gram‐positive and gram‐negative bacteria.

**Table 3:** Inhibition zone of the cinnamon extracts against foodborne pathogens

Extraction solvent	Cinnamon	Inhibition zone (*d*, mm)			
		*L. monocytogenes*	*S. aureus*	*E. coli*	*S. anatum*
N‐butane	*C. cassia*	37.45 ± 0.56	30.35 ± 0.45	28.87 ± 1.05	29.56 ± 0.73
	*C. loureiroi*	35.23 ± 0.98	25.86 ± 0.27	27.44 ± 0.37	33.76 ± 0.56
	*C. wilsonii*	21.86 ± 0.64	18.98 ± 0.72	20.33 ± 0.22	16.56 ± 1.18
	*C. burmannii*	24.32 ± 0.42	23.31 ± 0.84	20.61 ± 0.38	18.35 ± 0.57
Ethanol	*C. cassia*	8.77 ± 0.53	8.32 ± 0.53	7.67 ± 0.76	8.65 ± 0.35
	*C. loureiroi*	8.98 ± 0.80	10.11 ± 0.47	8.53 ± 0.34	9.67 ± 0.41
	*C. wilsonii*	7.85 ± 0.32	8.00 ± 0.54	7.11 ± 0.45	7.84 ± 0.38
	*C. burmannii*	8.00 ± 0.63	7.50 ± 0.39	7.43 ± 0.51	7.28 ± 0.17

The MBCs of cinnamon extracts were determined, and the results are listed in Table 4. N‐butane extracts of cinnamon were found much more sensitive to *L. monocytogenes*, *S. aureus*, *E. coli*, and *S. anatum* than ethanol extracts, especially *C. cassia* and *C. loureiroi*. The MBC values were ranged from 0.31 to 2.50 mg/ml for n‐butane extracts and 20.00 to 160.00 mg/ml for ethanol extracts. MBC values of N‐butane and ethanol extracts showed no significant differential between gram‐positive and gram‐negative bacteria which were in accordance with inhibition zone. N‐butane extracts of cinnamon had better antibacterial activities especially for *C. cassia* and *C. loureiroi* and more potential to be used as food biopreservatives than ethanol extracts.

**Table 4:** MBCs of the cinnamon extracts against foodborne pathogens

Extraction solvent	Cinnamon	MBC (mg/ml)			
		*L. monocytogenes*	*S. aureus*	*E. coli*	*S. anatum*
N‐butane	*C. cassia*	0.31	1.25	0.625	1.25
	*C. loureiroi*	0.62	1.25	0.625	0.62
	*C. wilsonii*	1.25	2.50	2.50	1.25
	*C. burmannii*	1.25	2.50	2.50	5.00
Ethanol	*C. cassia*	20.00	20.00	20.00	40.00
	*C. loureiroi*	40.00	80.00	80.00	80.00
	*C. wilsonii*	40.00	160.00	160.00	80.00
	*C. burmannii*	80.00	80.00	160.00	160.00

The antibacterial activity has been attributed to the presence of some active compounds in the extracts, and (*E*)‐cinnamaldehyde is known to inhibit bacterial acetyl‐CoA carboxylase and responsible for major antibacterial activity (Lopez, Sanchez, Batlle, & Nerin, 2007). The antibacterial action of (*E*)‐cinnamaldehyde is considered to obstruct the bacterial cell membrane and its structures which lead to ion leakage (Unlu, Ergene, Unlu, Zeytinoglu, & Vural, 2010). Eugenol and linalool also have been reported to have antibacterial activity in direct contact (Herman, Tambor, & Herman, 2016; Oyedemi, Okoh, Mabinya, Pirochenva, & Afolayan, 2009). (*E*)‐cinnamaldehyde has been proven to possess stronger antibacterial activity in comparison with eugenol and linalool (Mith et al., 2014), which could explain the higher antibacterial activity of *C. cassia* and *C. loureiroi* than *C. wilsonii* and *C. burmannii*.

Antibacterial activities of essential oil from *C. cassia* and *C. burmannii* also have been investigated (Abdelwhab et al., 2010). Broad‐spectrum antibacterial property was exhibited by essential oil which is in accordance with our research. Antibacterial activities of *C. loureiroi* and *C. wilsonii* extracts have not been reported according to the literature. Moreover, Shan et al. (2007) reported that the 80% methanol extract of *C. burmannii* exhibited antibacterial properties with much higher MBC (≥2,500 μg/ml) to *E. coli* and *S. aureus* than essential oil extracted by water (Awang et al., 2013). Gupta, Garg, Uniyal, and Kumari (2008) found the antimicrobial activities of *C. zeylanicum*'s essential oil were more potent than 50% ethanol extract. Mukhtar and Ghori (2012) demonstrated that antibacterial properties of ethanol extract of the cinnamon plant were more effective against both gram‐negative and gram‐positive bacteria than the aqueous extract. In consideration of extracts polarity, they were coincided with our study and can be deduced initially that the lower the polar extracts, the higher the antibacterial activity.

## 4 CONCLUSION

Subcritical n‐butane extracts of *Cinnamomum* species were much more sensitive to foodborne pathogens than ethanol extracts, especially for *C. cassia* and *C. loureiroi*. It can be attributed to the presence of the principle bioactive constituents, especially (*E*)‐cinnamaldehyde. Subcritical n‐butane extracts of *C. cassia* and *C. loureiroi* could be potential candidates to be used as natural alternatives for further application in food preservation to extend the shelf life of food products.

## CONFLICT OF INTEREST

The authors declare that they do not have any conflict of interest.

## ETHICAL STATEMENT

This study did not involve any human or animal testing. Moreover, human and animal testing was unnecessary in this study.
